# Spatial characteristics and economic value of threatened species (*Khaya ivorensis*)

**DOI:** 10.1038/s41598-020-63145-x

**Published:** 2020-04-14

**Authors:** Pasicha Chaikaew, Opeyemi Adeyemi, Adenule O. Hamilton, Omonu Clifford

**Affiliations:** 1 Department of Environmental Science, Faculty of Science, Chulalongkorn University, Bangkok, Thailand; 2 Environment, Health and Social Data Analytics Research Group, Chulalongkorn University, Bangkok, Thailand; 3 Department of Plant and Soil Science, University of Pretoria, Pretoria, South Africa; 4 Department of Forestry and Wood Technology, Federal University of Technology Akure, Akure, Nigeria; 5 Forestry Research Institute of Nigeria, Ibadan, Nigeria

**Keywords:** Biodiversity, Forestry

## Abstract

*Khaya ivorensis* (*K. ivorensis*), one of the most valuable tropical hardwood species indigenous to West and Central Africa, has been classified as a threatened tree species. However, information on its remaining population and distribution are limited. We mapped the current *K. ivorensis* spatial distribution, modelled the spatial autocorrelation and estimated its economic value using volume estimation and market pricing. The study was conducted in Odigbo and Irele local government areas (LGA), Ondo State, Nigeria. Spatially, localities of 97 *K. ivorensis* were identified across the study area and can be added into a wide range of datasets from local to global inventories. Large trees in diameter and height were statistically clustered in the north of Odigbo and assumed to relate with forest reserve management. Estimated median tree volumes were 0.39 m^3^ and 0.31 m^3^ in accordance with the allometric volume function and specific volume function, respectively. The economic values of wood varied approximately from US$111,208 to US$72,081. Findings from this study are a valuable resource for conserving this species and other threatened tree species.

## Introduction

Recent international agreements under Sustainable Development Goal 15 (Target 15.5 of the UN Climate Summit 2014) and the New York Declaration on Forests take ambitious goals to at least halve the rate of loss of natural forests globally by 2020 and take action to end natural forest loss by 2030, as well as protect and prevent the extinction of threatened species^1,2^. *Khaya ivorensis* (*K. ivorensis*), one of five species of African mahogany commonly found in coastal West Africa, Cote d’Ivoire through Ghana, and southern Nigeria to Cameroon, has been listed on the World Conservation Union (IUCN) Red List of Threatened Species since 1998^3^. With respect to IUCN criteria, this species meets the critical value for a threat status of A1 as ‘vulnerable’ and A2 as ‘near extinct’. *K. ivorensis* declined by 48% between 2005 and 2015 and is anticipated to reach 432% reduction in the next decade^4^.

While *K. ivorensis* remains an important tree species, it is widely sought after in international timber markets and used for a wide range of consumption such as veneer, interior joinery, exterior joinery, boat building, and medicinal purposes^5^. It is generally agreed that timber from man-made forests have lower yield and inferior wood quality compared to those from natural forests^6^. Mahogany plantation is no exception. High exploitation rates, combined with little or no regeneration after disturbance, has led *K. ivorensis* to become an endangered tree species. Furthermore, the plantation establishment of this species has been largely unsuccessful because of shoot borer *Hypsipyla robusta* Moore attack^7^. The shoot borer of these pest species destroys the leading shoot, resulting in poor form, and severe loss from stunted growth, which affect the quality and economic value of the timber^8,9^. The Global Forest Watch, University of Maryland, and World Resources Institute point out that of the ten countries ranked with the greatest percent increase in tree cover loss between 2001–2013 and 2014–2017, seven were marked in Africa, and one of these was Nigeria^10^.

Mapping tree positions at regional and local scales is required as a key indicator for establishing a baseline, tracking tree cover, and for global input. To our knowledge, the georeferenced ground data source for mapping *K. ivorensis* stands is still limited. The Global Biodiversity Information Facility (GBIF) recorded 35 occurrences of *K. ivorensis* in Nigeria from 1905–1976. The use of GBIF information can provide general background of physical occurrences of *K. ivorensis* from the availability of existing data portal; however, it is important to note that observation records contained in GBIF database have not been derived using consistent methodologies and might contain the error inherent in spatial data. In recent research, despite an unclear survey time period, 20 *K. ivorensis* stands were found in Ondo State^11^.

Up-to-date information on the occurrence and spatial characteristics of forest trees in an ecosystem can help address the concentrations of distribution and tree structures. In addition, estimating tree volumes from direct consumptive use can provide proxy economic values of *K. ivorensis* communities, which presented the value for the explicit use of forest; not necessarily the total value to nature. This study aims to identify current *K. ivorensis* spatial distribution with structural information and estimate the economic value based on the market price of the wood. Spatial characteristics and valuation of *K. ivorensis* may not provide a full basis for decision, yet they can be supplemental considerations to better support sustainable management schemes and conservation efforts.

## Study area

**Figure 1:**
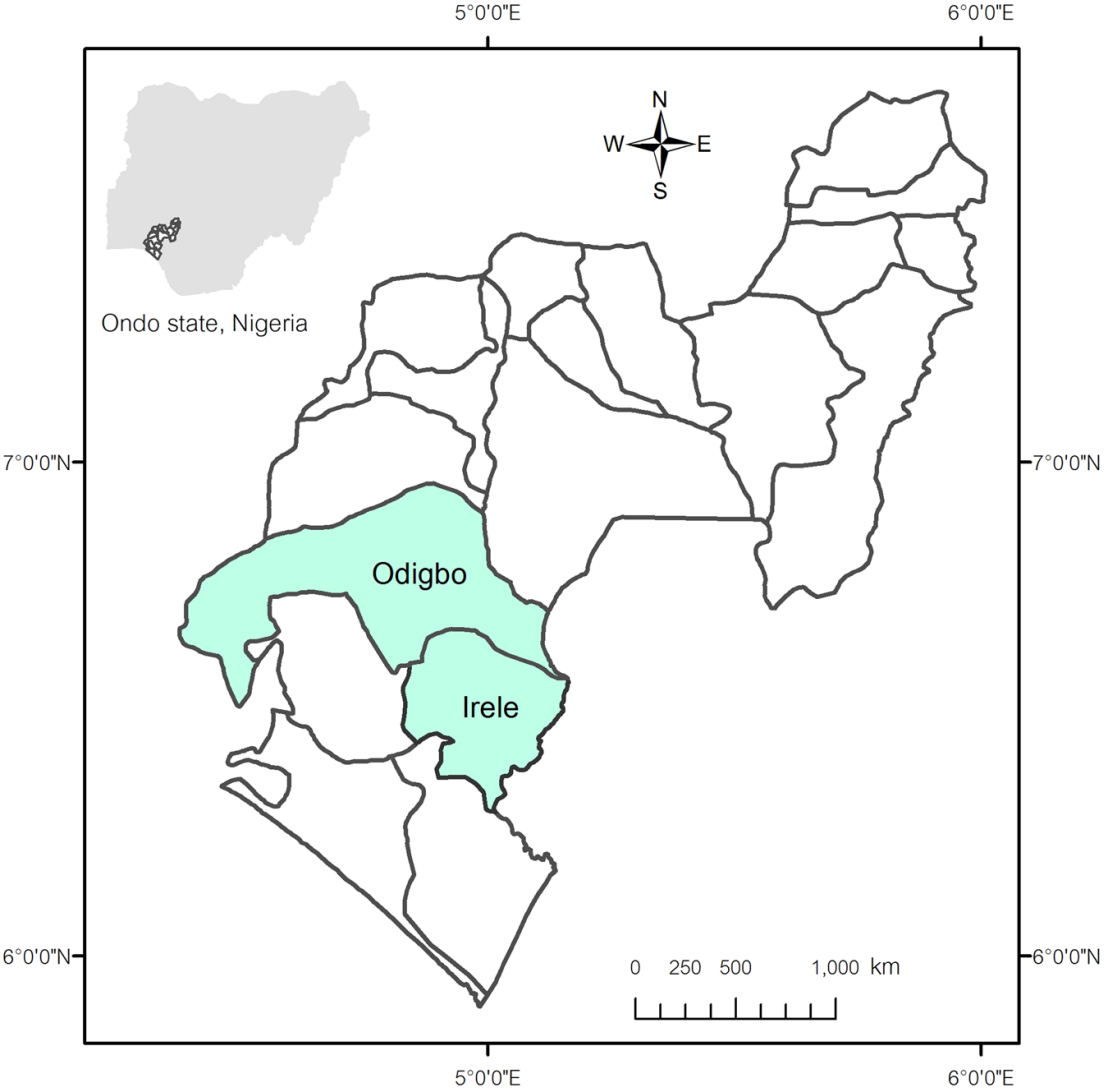
Map of the study areas; Odigbo and Irele local government areas within Ondo State, Nigeria.

This research was carried out in Odigbo and Irele LGA in the south of Ondo State, Nigeria (Fig. 1). Odigbo is bounded by latitudes 6°47′40″ N and longitudes 4°52′3″ E, with about 1,818 km^2^. Irele, an area adjacent to the southeast of Odigbo, is 963 km^2^ in area and bounded by latitudes 6°29′18″ N and longitudes 4°52′12.78″ E. Odigbo and Irele are lowlands within a humid forest zone with a mean cumulative annual rainfall of 1,320 mm. It has monthly mean temperatures that vary from 27.6 °C to 31.6 °C^12^. The wet season lasts about seven to eight months, while the dry season lasts three to four months^13^. Soil types are comprised of Dystric Nitosols (Nd) 57.42%, Ferric Luvisols (Lf) 22.26%, Eutric Nitosols (Ne) 11.87%, Gleysols (G) 7.64%, and Dystric Regosols (Rd) occupy 0.81% of the study area^14^.

## Results and discussion

### Spatial characteristics and distribution

**Figure 2:**
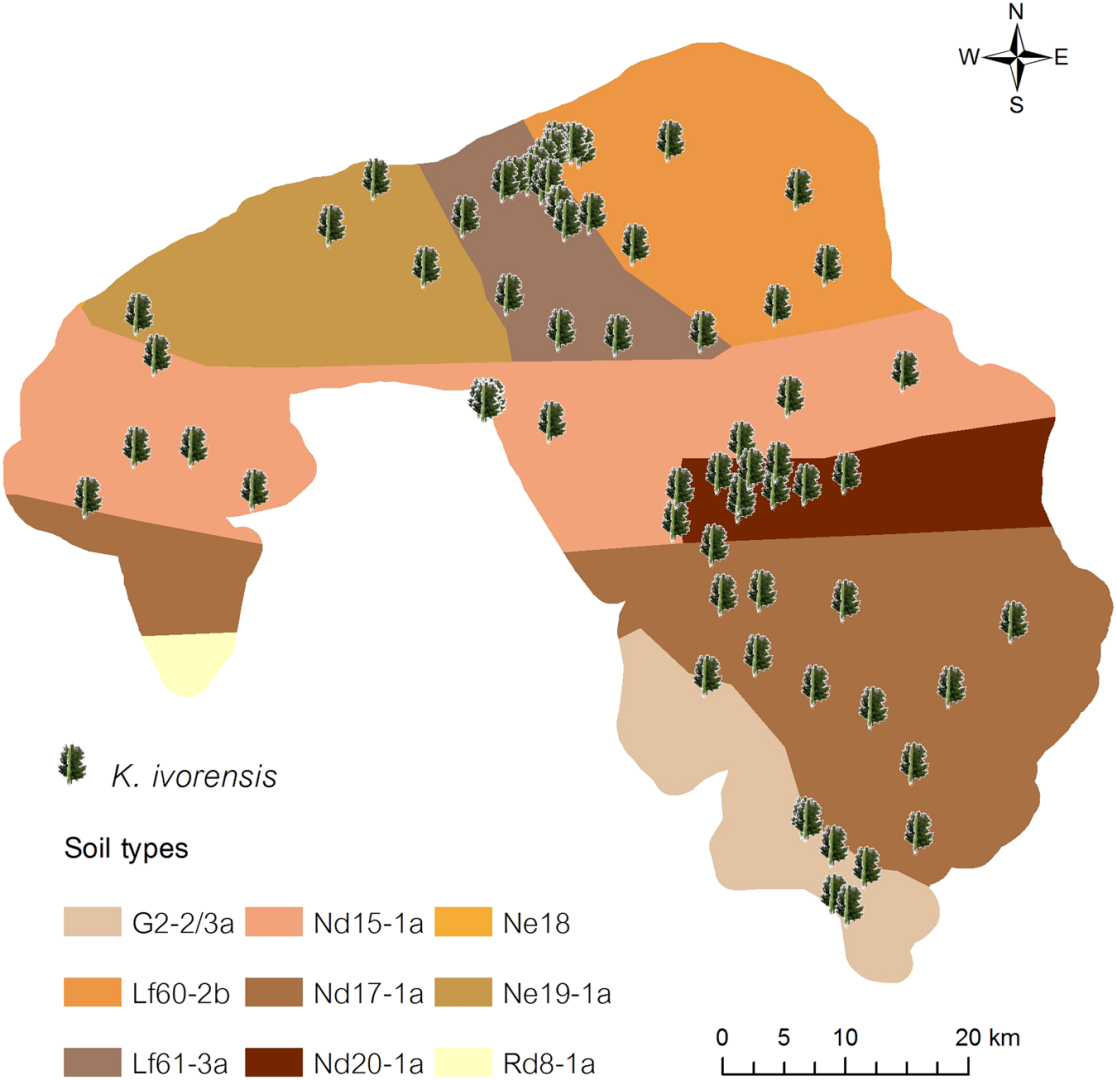
Spatial distribution of *K. ivorensis* on different soils in Odigbo and Irele local government areas.

A total of 97 *K. ivorensis* stands was recorded; 26 trees in Irele and 71 in Odigbo (Fig. 2). Their diameter at breast height (Dbh) ranged from 5 to 48 cm with a mean value of 18.9 cm, and height (Ht) ranged from 11 to 49.2 m with an average value of 24.9 m. The diameter at the middle (Dm) measurements were in between 3.5 and 34 cm with an average number of 13.5 cm. Odigbo and Irele LGA of Ondo State are known to be one of the habitats of *K. ivorensis* in southwest Nigeria where the species is more prominent. However, it was reported that the tree species is threatened with extinction. This study indicated that tree density per unit area was very low. Similar to Irele (0.02 tree/km^2^), Odigbo experienced 0.03 tree/km^2^ density. More *K. ivorensis* trees were observed during our survey as compared to Lawal *et al*.^11^ study in Ondo state and the GBIF record^15^. The findings can be added into a wide range of datasets from local to global inventories. Geographic locations can enhance remote sensing techniques for further conservation of this species. As graphically explained in Fig. 2, *K. ivorensis* can grow across G, Nd, Ne, and Lf soil types. In Malaysia, *K. ivorensis* was introduced as one of eight promising exotic tree species for large-scale forest plantation. Heryati *et al*.^16^ discovered the high survival rate (≥94%) of five-year old *K. ivorensis* planted in Ultisols and suggested that a land-clearing environment was suitable for this species.

**Figure 3:**
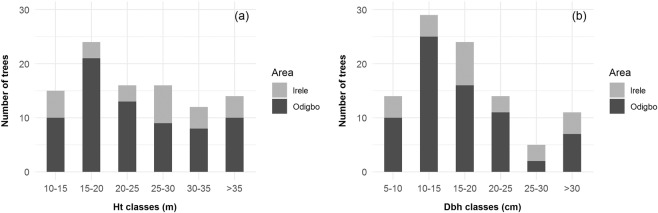
Distribution of (**a**) height and (**b**) diameter at breast height of *K. ivorensis* in Irele and Odigbo local government areas.

Similar to other African mahogany, *K. ivorensis* growth factors depend directly on the soil, solar radiation, and aspects of latitude. Trees can grow quickly and produce high-quality timber in hygrophilous evergreen forests and soils with reduced water retention capacity^17^. Trees in natural habitats can grow up to 40–50 m in height and 2 m in diameter^18^. Our study sorted Dbh and Ht into six classes as shown in Fig. 3. Even though Lawal *et al*.^11^ reported that more populations of *K. ivorensis* were found in the 10–15 m height class, our study found that most trees were in the 15–20 m height class. While the population was generally few in all Dbh classes in southwestern Nigeria^11^, our survey indicated more trees fell in second- and third-diameter classes (10–15 cm and 15–20 cm). The observations implied a positive stance of forest conservation in this area. Occurrence surveys and species monitoring are suggested to other *K. ivorensis* habitats. Further sustainable land management and protection are also recommended for *K. ivorensis* conservation of concern.

Considering the temporal limitation of this study, the mean annual growth increment in diameter and height have been reported in other studies. In Nigeria, the record showed that the average height of trees after four years was 4.5 m with a trunk diameter of 8 cm, which accounted for 1.12 m year^−1^ in height and 2 cm year^−1^ in diameter of average annual growth^19^. In Cote d’Ivoire, mean annual growth increments of four-year-old *K. ivorensis* were 2.3 m year^−1^ in height and 2.5 cm year^−1^ in diameter^18^. Growth performances of the *K. ivorensis* plantation in Malaysia, on the other hand, varied across soil series. Mean annual height increment of five-year-old *K. ivorensis* trees ranged from 1.57 to 2.11 m year^−1^ and mean annual diameter increments varied from 2.32 to 2.88 cm year^−1^ ^16^.

### Spatial autocorrelation and hot spot analysis

The results of the spatial autocorrelation based on the Euclidean distance method suggested that the Dbh, Ht, and Dm patterns of *K. ivorensis* in the study area were clustered with different magnitudes (Table 1).

**Table 1:** Global Moran’s I summary of diameter at breast height (Dbh), height (Ht), and diameter at middle (Dm) of *K. ivorensis* spatial distribution.

	Moran’s I Index	z-score	p-value	Interpretation
Dbh	0.305	2.634	0.008	Less than 1% likelihood that this clustered pattern could be the result of random chance
Ht	0.228	1.981	0.048	Less than 5% likelihood that this clustered pattern could be the result of random chance
Dm	0.206	1.811	0.070	Less than 10% likelihood that this clustered pattern could be the result of random chance

After the clustering pattern of *K. ivorensis* was confirmed by the Moran’s I statistic, the Getis-Ord ${G}_{i}^{\ast }$ statistic was applied to measure the intensity of clustering of high or low values in a Gi bin relative to neighbouring bins within an identified cube. A high z-score and small p-value for *K. ivorensis* indicated a spatial clustering (hot spot) of healthy trees in terms of physical structures based on Dbh and Ht, while a low negative z-score and small p-value identified a spatial clustering (cold spot) of small tree structure. A hot spot was depicted by a 95% confidence interval which showed that there were 20 trees of large diameter and height in the northern area of Odigbo. A cold spot identified by 32 trees of small size was described with a 95% confidence interval. Of 32 trees, 31 were marked in Odigbo and one in Irele. The remaining 45 *K. ivorensis* stands showed no apparent spatial clustering or structural variations. The existence of similar tree sizes in the same area may respond to the indicators of the site conditions. Major factors influencing survival rate such as the light intensity, moisture, soil, spacing, and (micro) climatic conditions were reported in other studies^16,20,21^.

**Figure 4:**
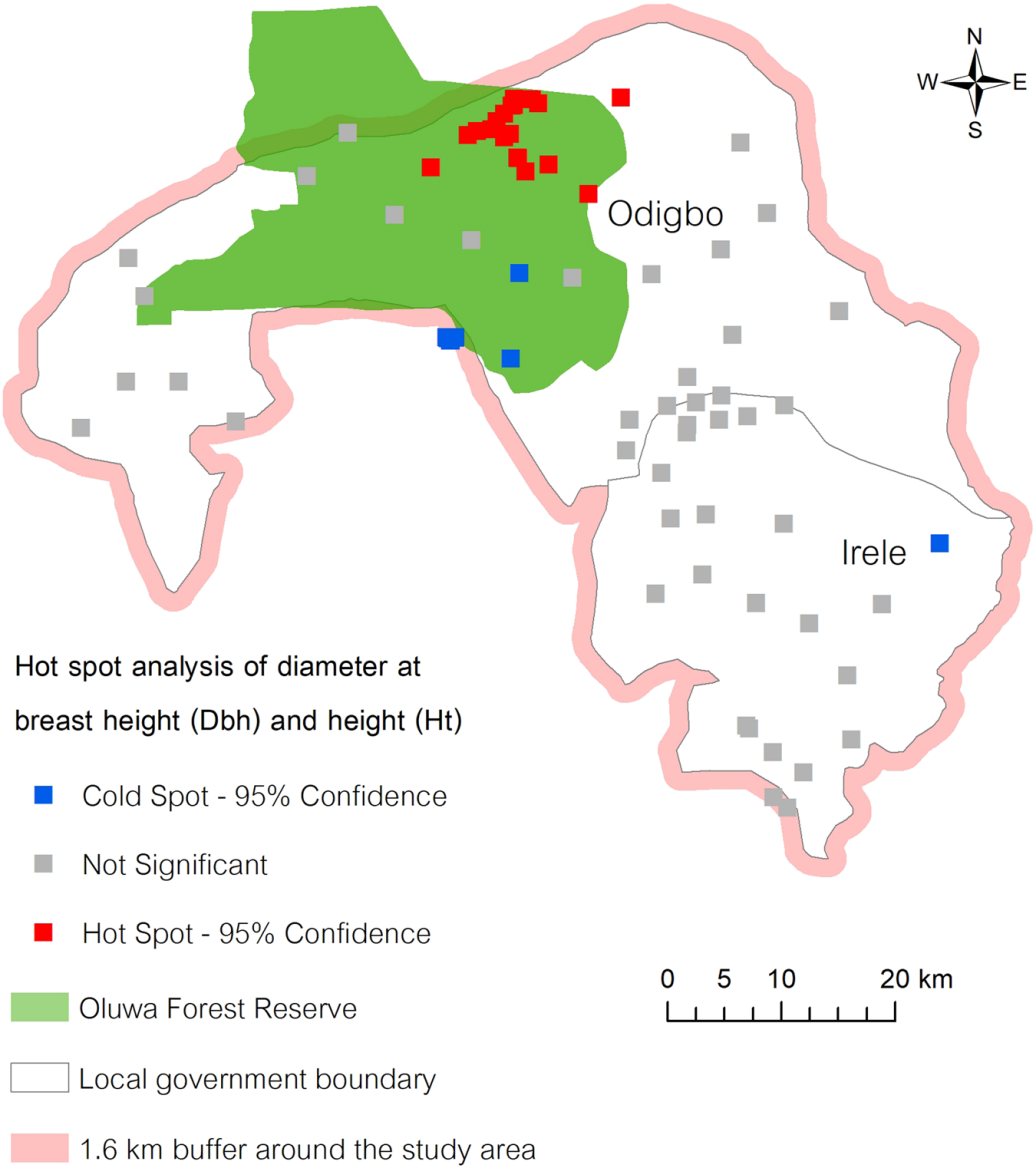
The result of point-based hot spot analysis. Hot spots (in red) represent statistically significant clustering of large diameter trees at breast height (Dbh) and height (Ht) of *K. ivorensis* stands, while cold spots (in blue) show statistically significant clustering of small trees’ Dbh and Ht.

In addition, it was observed that hot spots fell in the northern region of Odigbo within the Oluwa Forest Reserve (Fig. 4)^22^. The Oluwa Forest Reserve is part of three large contiguous forest reserves – Omo (Ogun State), Shasha (Osun State), and Oluwa (Ondo state) – known as the Omo-Shasha-Oluwa forest complex^23^. This may reflect the important role of forest reserves as an effective conservation landscape for this species. In contrast, a high level of anthropogenic activities combined with intense farming activities and several settlements were observed in Irele during the survey work. These activities could be one reason for fewer *K. ivorensis* in this area. Such activities could have a significant impact on biodiversity, and thus, demand urgent intervention. The spatial assessment taken in this study not only informs conservation stakeholders where to locate *K. ivorensis*, but also supports an intentional effort towards conserving the species.

### Volume estimation and economic value

Volume functions applied in this study included Dbh and Ht as measurement predictors. According to the allometric volume equation, the total volume of *K. ivorensis* trees within Odigbo and Irele was 48.05 m^3^. The median estimated volume was 0.39 m^3^ with mean ± SD of 0.76 ± 1.00 m^3^. Due to comparable soil types, that is, Humic Gleysol and ferric Luvisols, and similar humid equatorial climatic conditions for *K. ivorensis* growth, another specific function selected for this study was based on the prediction model developed in Minas Gerais state, Brazil^24^. Two classes were separated for this study estimation: first thinning (Dbh <30 cm) and final cut (Dbh ≥ 30 cm). The double-entry equations presented a total volume of 74.14 m^3^ across the study area. The median value was 0.31 m^3^ with mean ± SD of 0.50 ± 0.54 m^3^.

**Figure 5:**
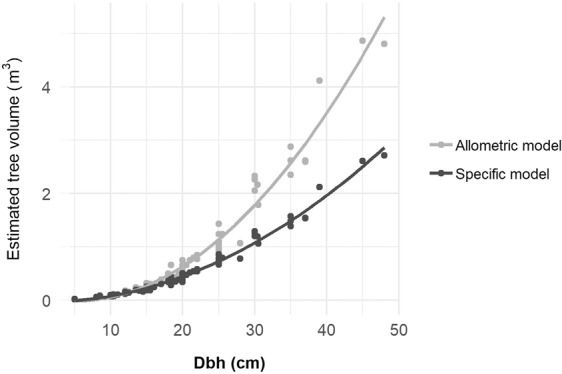
Quadratic relationships between tree volume and Dbh derived by the allometric volume function from Eq. 3 and the specific volume function from Eq. 6.

Tree volumes were associated with Dbh in a quadratic pattern obtained by both estimated functions (Fig. 5). The parameters of the best-fit models for estimating commercial tree volume as a function of Dbh were statistically significant with r^2^ = 0.97 (p < 0.001) from the allometric volume function and r^2^ = 0.99 (p < 0.001) from the specific volume function. Applying the allometric models for tree volume in a tropical humid forest in Costa Rica, the power trend line pattern was discovered between stem volume and Dbh (r^2^ = 0.66) and total tree volume and Dbh (r^2^ = 0.81)^25^. The allometric volume function is considered a generalisation model for all sizes of trees. It can be noticed in Fig. 5 that the discrepancy volume values between both equations were greater when trees became larger. While inclusion of tree classes based on diameters with transformations of specific formulas for each class showed very strong prediction. The specific function is therefore recommended to estimate *K. ivorensis* volume with inclusion of other observed parameters such as tree age and the diameters of two ends and the middle. Most importantly, to further validate the model, observed tree volume is required.

*K. ivorensis* is well-known for its highly valuable timber in the international market. In local and regional context, tree valuation holds great potential for conservation that can be cooperated with market-based incentive policy. The International Tropical Timber Organization (ITTO)^26^ reported that the average price for 1 m^3^ of *K. ivorensis* was US$1,500. Applying this price, the approximation of wood values estimated using different volume functions would be equivalent to US$111,208 based on the allometric volume function and US$72,081 based on the specific volume function. Total economic value changes of the tropical forest biome in Africa are expected to decrease by €9,217 million in 2050 with respect to the baseline year 2007 (€1 = US$1.12 as of July 2019) or about 22% decrease of forest ecosystem services in terms of economic valuation^27^.The use of market-based instrument can align with a land management policy and public education to provide conservation actions. Other than timber, *K. ivorensis* provides other ecosystem services such as climate regulation, enhanced soil retention, improved water purification, pollination, landscape aesthetics, and habitats for variety of species^27^. The global reach to protect forests has been developed through market forces driven by the REDD + (Reducing Emissions from Deforestation and Forest Degradation). Financial mechanisms involve carbon emissions trading through forests and financial rewards to developing countries for effective forest management. The intrinsic value of forests provide a great potential to the weight given to conserving forests in the decision-making process^28^. Although there is no one-size-fits-all strategy for conserving *K. ivorensis* species, outcomes from this study can benefit the inventory of the global conservation status of the threatened species and improve the conservation efforts and help ensure the better use of such values in policymaking and in the decision among land-use options.

## Methods

### Tree data collection

The locations of the existing trees were collected as part of a group interview of farmers, tree finders, members of village community, and other key informants who have the extensive knowledge of tree identification. The data obtained from knowledgeable human resources were coped with ground survey from a team of local forestry experts. Forestry experts assisted during the inventory exercise of the existing *K. ivorensis* presence in the wild forest and free areas between July and September 2017. The ground survey was conducted in a systematical approach in each village from north to south. The field work involved taking the geographical coordinates of each *K. ivorensis* tree with the use of a handheld geographic positioning system (GPS) device (Garmin GPS 60) within Odigbo and Irele government areas. The reference system used for GPS positioning was the World Geodetic System (datum WGS84). The coordinates of each of the trees with identification numbers were added to the map to display their positions. Additional tree structure data such as Dbh, Dm, and Ht of each tree were measured. The manual readings of the Dbh through the circumference were taken by using a girth tape at 1.37 m above ground level. Tree Ht and Dm were measured using a telescopic Spiegel-Relaskop.

### Spatial analysis and data management

Descriptive statistics of the tree characteristics were performed across the selected area. All the pre-processing steps for spatial analysis of *K. ivorensis* coordinates acquired for this study were first geo-referenced and then reprojected with UTM WGS84 projection zone 31. A 1.6 km buffer zone was created to cover the tree observations adjacent to the study area. All spatial analysis was computed using ArcGIS® software v.10.6.1.

### *K. ivorensis* distribution and spatial autocorrelation

In addition to the explicit spatial locations of trees, this study applied Global Moran’s I technique, which is commonly used as an indicator of spatial autocorrelation^29^. Moran’s I reflects the correlation of the spatial relationship among observations in a neighbouring pattern computed by the statistic Moran’s I index (Eq. 1).

1 $$I=\frac{N{\sum }_{i=1}^{n}{\sum }_{j=1}^{n}{w}_{ij}({x}_{i}-\bar{x})({x}_{j}-\bar{x})}{({\sum }_{i=1}^{n}{\sum }_{j=1}^{n}{w}_{ij}){\sum }_{i=1}^{n}{({x}_{i}-\bar{x})}^{2}}$$

where *N* is the number of observed trees; $\bar{x}$ is the mean of the variable of the whole region; *x*_*i*_ is the value of the variable at a particular location *i*; *x*_*j*_ is the value of the variable at other locations; *w*_*ji*_ is the distance weighting between locations *x*_*i*_ relative to *x*_*j*_.

Moran’s I index values range from −1 to 1. A high positive index suggests that a target variable value is similar to its neighbourhood. This implies spatially autocorrelated variables among locations, which include high-high clusters and low-low clusters. A negative index value indicates that the spatial variable distribution of high and low values is more spatially dispersed. When Moran’s I approaches zero, spatial randomness is expected. The null hypothesis for the Global Moran’s I states that the spatial attribute being analysed is randomly distributed among the neighbouring features in the study area. When a z-score is larger than 1.96 or lower than −1.96 (p < 0.05), the null hypothesis is rejected and implies that the spatial autocorrelation is significant. The Global Moran’s I has been widely used in the forestry field to study spatial patterns of natural forest growth and plant populations^30,31^, species-based similarities^32^, spatial differentiation characteristics, and driving forces of forest transition^33^. In this study, the spatial autocorrelation of Dbh, Dm, and Ht was analysed.

### Hot spot detection

Hot spot analysis has been used to pinpoint priority areas for forest protection and conservation^34–37^. The fundamental Getis-Ord ${G}_{i}^{\ast }$ statistic measures the intensity of clustering of high or low values^38^, which can identify clustering of spatially local phenomena. A simple form of the ${G}_{i}^{\ast }$ statistic can be written as^39^:

2 $${G}_{i}^{\ast }=\frac{{\sum }_{j=1}^{n}{w}_{ij}{x}_{j}}{{\sum }_{j=1}^{n}{x}_{j}}$$

where ${G}_{i}^{\ast }$ is the statistic that describes the spatial dependency of location *i* over all *n* locations; *x*_*j*_ is the magnitude of the variable *x* at event *j* over all *n*; *n* is equal to the total number of features; *w*_*ij*_ is the weight value between location *i* and *j* that represents their spatial interrelationship. The outcomes express the z-score and p-value of the computed ${G}_{i}^{\ast }$ in comparison with the data normal distribution. This study applied point-based hot spot analysis on the Dbh and Ht across Odigbo and Irele LGA. When a z-score of ${G}_{i}^{\ast }$ is larger than 1.96, the point can be considered as a hot spot (high values) at the confidence level of 95%, if smaller than −1.96, that point is considered a cold spot (low values).

### *K. ivorensis* economic value

Forests deliver multiple ecosystem services, namely timber and fuelwood, fruits and honey, biodiversity conservation, carbon sequestration, wilderness, erosion control, nutrient cycling, scenic beauty, and spiritual values^40^; however, this study is limited to estimation of the economic value of *K. ivorensis* based on consumptive timber use. Two main factors are taken into value estimation: tree volume and wood price.

Volume functions can be categorised into three classes: first, using Dbh as a sole predictor (local volume functions); second, having Dbh and Ht measurements as predictors (regional volume functions); and third, including Dbh, Ht, and an upper stem diameter as a surrogate for stem form, which is particularly good at the national scale (large-scale volume functions)^41^. Oliveira *et al*.^24^ suggested that double-entry models (Dbh and Ht predictors) are more accurate than single-entry models (Dbh predictor) when predicting *K. ivorensis* stand volume. This study adopted two approaches for indirect volume functions: the allometric volume equation (Eq. 3)^42^ and the specific *K. ivorensis* volume equation obtained by Oliveira *et al*.^24^ (Eqs. 4 and 5).

3 $${V}_{A}=\sum (\frac{Db{h}^{2}}{4}\ast Ht\ast \pi \ast {f}_{i})$$

where *V*_*A*_ is tree volume determined by allometric equation; *Dbh* is tree diameter at breast height (cm); *Ht* is the total height of the tree (m); *f*_*i*_ is the form factor of the tree depending on the shape of the tree species (0.7)^42^.

Specific *K. ivorensis* volume equations were more complicated since they were calculated from different models; then compared with observed volumes from Smalian’s formula for validation of the results. First thinning and final cut were estimated separately with respect to tree ages. Tree ages, however, were unknown for this study, Dbh of <30 cm and ≥ 30 cm were thus presumed to define the first thinning and final cut, respectively. Oliveira *et al*. [26] proposed the Schumacher and Hall model^43^ as the best equation to estimate consumptive volume in Minas Gerais stands, where soils and climate are comparable to our study area. Formulas are written as follows:

4 $$V{s}_{first}=0.0002428\ast Db{h}^{1.849}\ast H{t}^{0.5952}$$

5 $$V{s}_{final}=0.0001452\ast Db{h}^{1.962}\ast H{t}^{0.6165}$$

6 $$V{s}_{total}=V{s}_{first}+V{s}_{final}$$

where *Vs*_*first*_ is the estimated first thinning of *K. ivorensis* volumes (m^3^); *Vs*_*final*_ is the estimated final cut of *K. ivorensis* volumes (m^3^); *Vs*_*total*_ is the sum of the first thinning and final cut volumes (m^3^).

The value of provisioning ecosystem services, such as timber, can be estimated using market values^40^. The market price for *K. ivorensis* timber value varied from US$500 to US$1,500 per cubic meter. For the economic value, US$1,500 was applied.

## Supplementary information

Supplementary information
